# PARTICIPATORY DESIGN: AN ESSENTIAL PROCESS FOR SOCIALLY ASSISTIVE ROBOTIC ACTIVITIES IN LONG-TERM CARE SETTINGS

**DOI:** 10.1093/geroni/igac059.1661

**Published:** 2022-12-20

**Authors:** Mion Lorraine, Emily Latshaw, Yi Chun Lin, Miroslava Migovich, Ritam Ghosh, Nibraas Khan, Nilanjan Sarkar, Judith Tate

**Affiliations:** The Ohio State University, Columbus, Ohio, United States; The Ohio State University, Columbus, Ohio, United States; The Ohio State University, Columbus, Ohio, United States; Vanderbilt University, Nashville, Tennessee, United States; Vanderbilt University, Nashville, Tennessee, United States; Vanderbilt University, Nashville, Tennessee, United States; Vanderbilt University, Nashville, Tennessee, United States; Ohio State University, College of Nursing, Columbus, Ohio, United States

## Abstract

We conducted participatory design research for long term care (LTC) socially assistive robotic activities comprised of social, cognitive and physical components and enhanced human-robot (HRI) and human-human interactions (HHI). Repeated sessions were conducted with 10 geriatric experts (physicians, activity directors, nurses, occupational therapist) and 12 LTC residents (ages 70–92). Two robots, animal and humanoid, were used in combination with virtual reality. Four collaborative activities for paired older adults were designed and evaluated: playing drums to music, completing paintings, a fishing game, and training a dog with simple commands. Within each activity, three levels of difficulty were designed. Stakeholder feedback was obtained through observations and interviews. Numerous modifications were made following each session that addressed hardware, software and activity issues. Modifications were necessary both for the HRI and HHI aspects of the activity. Our experience demonstrates the necessity for participatory design in the deployment of technology for LTC settings.

